# Statistical learning and the uncertainty of melody and bass line in music

**DOI:** 10.1371/journal.pone.0226734

**Published:** 2019-12-19

**Authors:** Tatsuya Daikoku

**Affiliations:** 1 Department of Neuropsychology, Max Planck Institute for Human Cognitive and Brain Sciences, Leipzig, Germany; 2 Centre for Neuroscience in Education, Department of psychology, University of Cambridge, Cambridge, United Kingdom; Nottingham Trent University, UNITED KINGDOM

## Abstract

Statistical learning is the ability to learn based on transitional probability (TP) in sequential information, which has been considered to contribute to creativity in music. The interdisciplinary theory of statistical learning examines statistical learning as a mechanism of human learning. This study investigated how TP distribution and conditional entropy in TP of the melody and bass line in music interact with each other, using the highest and lowest pitches in Beethoven’s piano sonatas and Johann Sebastian Bach’s Well-Tempered Clavier. Results for the two composers were similar. First, the results detected specific statistical characteristics that are unique to each melody and bass line as well as general statistical characteristics that are shared between the melody and bass line. Additionally, a correlation of the conditional entropies sampled from the TP distribution could be detected between the melody and bass line. This suggests that the variability of entropies interacts between the melody and bass line. In summary, this study suggested that TP distributions and the entropies of the melody and bass line interact with but are partly independent of each other.

## 1. Introduction

### 1.1. Statistical learning in humans and computers

Statistical learning (SL) has been considered a domain-general and implicit learning system that encodes probabilistic distribution of sequential phenomena such as music and language [1–3]. For example, the brain’s SL machinery automatically computes transitional probability (TP) distributions of sequences, calculates uncertainty/entropy of the distribution, and predicts a future state based on an internalized statistical model in order to minimize sensory reaction and uncertainty and optimize the efficiency of the prediction. SL is an interdisciplinary field that embraces both the brain’s SL system and artificial intelligence in the framework of predictions. When a brain or a computer encodes the TP distribution of a sequence, it expects a probable future stimulus with a high TP and inhibits the processing loads that will arise in response to predictable states [4][5]. SL has been considered to contribute to creativity in music [6,7], decision-making [8–10], and motor activities [11,12][13] as well as perception [14,15][16,17]. The TP is a conditional probability of an event B given that the latest event A has occurred, written as P(B|A). The TP distributions sampled from sequential information can be expressed by nth-order Markov models or n-gram models [18]. The Markov model has frequently been applied to develop artificial intelligence that gives computers learning abilities similar to those of the human brain, thus generating systems for data mining, automatic music composition [19], and automatic text classification in natural language processing [20].

Psychologists agree that computational and corpus studies on music can highlight some of the statistical properties available to musical learners by SL and implicit learning [21–24]. Particularly, the Competitive Chunker [25], PARSER [26], Information Dynamics of Music (IDyOM) [27], and n-gram models [28] underlie the hypothesis that music is acquired by concatenating chunks. Computational studies calculate statistical distributions in music and devise corresponding models, then evaluate the validities of these models through neurological and behavioural experiments [27,29,30]. Particularly, SL in Markov models, which correspond to n-gram models based on conditional probability [31], overlaps with SL in many other fields of study, such as neuroscience, behavioural science, and computational science. Entropy, which is calculated from the probability distribution and has been interpreted as the average degree of surprise associated with an outcome [32,33], has also been used to verify the validity of computational models including SL in music [34–37]. Thus, information-theoretical approaches including information content and entropy (i.e., transitional probability and uncertainty, respectively) based on n-order Markov models are candidates for understanding musical SL on an interdisciplinary scale.

### 1.2. Uncertainty, probability, and order

To precisely predict individual events in a sequence, the brain encodes the degree of uncertainty of the statistical distributions in the sequence as well as the TP value itself [34,38]. This uncertainty can be evaluated using “*entropy*” as Shannon has done [31]. Particularly, conditional entropy can be calculated from TP distribution, interpreted as the average degree of surprise or uncertainty of an outcome. From a psychological perspective in music, a musical sequence with higher conditional entropy is considered to have information that makes its distributional structure more difficult to grasp. Therefore, in terms of information efficiency, an SL model sampled from a sequence with higher conditional entropy will be less optimized. Several studies have shown that the degree of conditional entropy modulates the precision of predictability in a sequence [30,39–41]. In addition, the uncertainty in musical sequences may account for the characteristics of musical SL ability in persons with developmental learning disorders such as amusia [42–44]. The literature on this topic indicates that persons with developmental learning disorders are impaired only with regard to higher- rather than lower-order SL [45]. Computational modelling has also suggested that individual differences in statistical knowledge gradually emerge from the lower- to higher-order SL models [46][47], and that statistical knowledge may shift from a lower- to higher-order (deeper) hierarchy through experience. Thus, distinct stages of SL strategies may be explained based on the information-theoretical concept of “*order*”. The order of SL is not independent of but rather interdependent on the degree of uncertainty[48]. In the framework of information theory, higher-order statistical models represent lower conditional entropy (i.e., uncertainty) (see Fig 3B in [18]). In other words, when the brain can construct a higher-, but not a lower-, order statistical model from music, it can internalize the music as having less uncertainty. Thus, the order of the SL model in music could modulate the uncertainty.

### 1.3. Creativity and uncertainty

Recent literature has suggested that specific developmental processes modulate SL ability in the brain. For example, both Western-classical and jazz musicians are better statistical learners in general than nonmusicians [49–53]. Furthermore, through long-term musical training, musicians optimize their brains’ probabilistic modelling ability for SL and decrease the degree of uncertainty [52]. In the end, the optimized SL models in musicians’ brains allow them to precisely and efficiently predict tones during SL of auditory sequences. This precision and efficiency of prediction may also enhance neural-processing efficiency. For example, neurophysiological studies have demonstrated the existence of individual differences in SL ability in the framework of prediction [54]. This may indicate that auditory training modulates neural processing that may reflect prediction based on SL. Although the brain tries to realize valuable behaviours at the lowest uncertainty, it also seeks a slightly suboptimal solution if such a solution can be afforded at a significantly low uncertainty [55]. This fluctuation of uncertainty could contribute to maximizing the rewards of curiosity, encouraging human creativity and creating new information regularities [56]. Recent computational studies on music have suggested that, from the early stage to the late stage of a composer’s lifetime, the transitional probabilities of familiar phrases in that composer’s music gradually decrease [46], whereas the conditional entropy (i.e., uncertainty) gradually increase. These findings were more prominent in higher- than in lower-order SL models. These studies suggest that higher- rather than lower-order statistical knowledge [46][38] may be more susceptible to long-term experience that modulates uncertainty in the brain’s probabilistic model [52]. Furthermore, computational studies on improvisation in music have suggested that lower-order SL models represent general characteristics shared among musicians, whereas higher-order SL models detect specific characteristics unique to each musician [57][58]. Thus, a growing body of literature indicates that SL affects musical structure and its statistical distributions. It is unknown, however, how the TP distributions of the melody and bass line interact with each other, and how tonal mode and key govern the statistical distributions and the interactions between the melody and bass line.

Western tonal classical music has a number of specific features such as isochronic metrical grids, tonal pitch spaces, hierarchical tension, and attraction contours based on the structure of the melody and chord progression [59,60]. The musical melody and bass line can interact with each other within the constraints of these features. In music, the highest and lowest pitches play an important role in establishing the frames of the melody and bass line, respectively. To form musical structures such as phrase and harmony, they are partly dependent and partly independent of each other. According to neurophysiological and behavioural studies, SL of dyad sequences with distinct regularities in each high and low voice can be performed in parallel and independently [61,62]. In other words, distinct statistical knowledge of high- and low-pitch sequences can be acquired simultaneously. Another neurophysiological study suggested that SL is also possible for harmony sequences in which the highest and lowest pitches are randomly distributed without regularity [29]. Together, neural studies support the hypothesis that SL of the melody and SL of the bass line interact with and are partly independent of each other in the framework of the Gestalt principle in music [60]. To understand musical SL in humans and to refine the computational models, it is important to examine how the melody and bass line interact with each other based on statistical and music-specific features.

### 1.4. The aim of the present studies

The purpose of the present studies is to investigate how TP distributions of the melody and bass line interact with each other, and how tonal mode and keys govern the statistical distributions and the interaction between the melody and bass line. The information content of TPs in the sequences containing the highest and lowest pitches in all of the movements in Beethoven’s piano sonatas (No.1, Op.2-1 to No.32, Op.111) (Study 1) and Johann Sebastian Bach’s Well-Tempered Clavier (Study 2) were calculated based on six different order Markov stochastic models (i.e., zeroth- to fifth-order Markov chains). First, to investigate the statistical characteristics of the melody and bass line in each piece of music, the TP distribution was analysed using principal component analysis, based on the hypothesis that there are fundamental statistical characteristics shared between the melody and bass line, and specific statistical characteristics that are unique to each. Additionally, the detectability of these characteristics may depend on the tonal mode and the keys [63] and/or on the order of TP distributions (first to sixth orders). If so, the interaction of statistical characteristics between the melody and bass line may depend on the tonality (tonal mode and keys) and/or order of the TP distribution[64]. Second, to investigate the relationships between entropy in the melody and entropy in the bass line in each tonality and each order of TP distribution, the conditional entropy of the TP distribution was compared by correlation analysis between the melody and bass line, and between music in a major key and music in a minor key. It was hypothesized that the variability of entropy in each piece of music depends on the tonality and order of TP distribution. In the present studies it was expected that the statistical distribution of music would correspond with models of predictive function in the brain, and we first investigated how information-theoretical notions including information content and entropy are related to SL theory regarding human predictions.

## 2. Methods

All of the movements in Ludwig van Beethoven’s piano sonatas (No.1 in F minor, Op.2-1 to No.32 in C minor, Op.111, composed 1795–1822) and Johann Sebastian Bach’s Well-Tempered Clavier, BWV 846–893, which is a collection of two series (No.1 and No.2) of preludes and fugues in all 24 major and minor keys, were used in the present studies. Using a scorewriter software program (Finale version 25, MI Seven Japan, Inc.), electronic scoring data of the sequences of highest pitch were extracted from the XML files. The highest and lowest pitches were defined as the highest and lowest pitches that can be played at a given point in time; in identifying these pitches, equivalent pitches were counted as one, and grace notes were excluded. Using all the pitch sequences in each piece of music, the TPs distributions were calculated based on zeroth- to fifth-order Markov models. In Beethoven’s piano sonatas, the weighted averages of TPs of all the movements were calculated. In Bach’s Well-Tempered Clavier, the weighted averages of TPs of the prelude and fugue in No.1 and No.2 in each key were calculated. As described in detail previously [57], the *n*th-order Markov models are based on the conditional probability of an element e_n+1_, given the preceding *n* elements:

$$P(e_{n+1}|e_{n})=\frac{P(e_{n+1}\cap e_{n})}{P(e_{n})}$$ (1)

**Fig 1:**
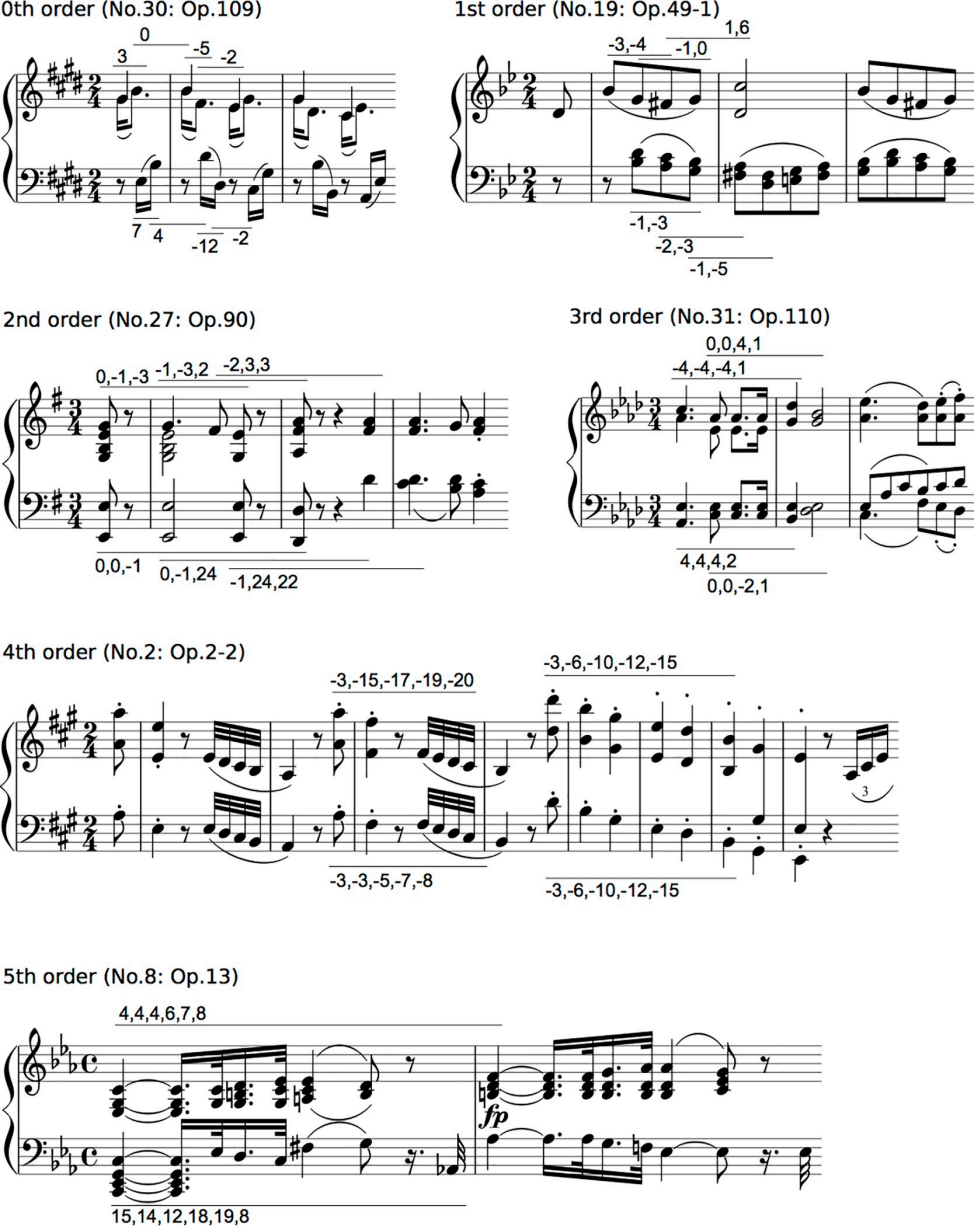
Representative phrases of transition patterns in the melody and bass line from zeroth- to fifth-order Markov models (Beethoven’s piano sonata).

Then, for each type of pitch-interval transition, all of the intervals were numbered so that an increase or decrease in a semitone was 1 or -1, respectively, based on the first pitch. Representative examples are shown in Fig 1. This revealed interval patterns but not pitch patterns. This procedure was employed to eliminate the effects of key changes on transitional patterns. The interpretation of a key change depends on the musician and is difficult to define in an objective manner. Thus, the results of the present studies may represent a variation of statistics associated with relative pitch rather than absolute pitch. Then, the information content (*I[e*_*n +1*_*|e*_*n*_*]*) in each TP was calculated based on information theory [31] as:

$$I(e_{n+1}|e_{n})={log}_{2}\frac{1}{P(e_{n+1}|e_{n})}(bits)$$ (2)

The SL mechanism can be explained using well-defined principles of information theory [31]. Information, also referred to as information content, is measured in binary integers or bits. The key insight is that information, i.e., the sum of the bits required to transmit a message, has entropy, i.e., “*uncertainty*” of statistical distribution. Thus, using the distributions of TPs (information content) in each melody and bass line of each piece of music, the distributional characteristics of each piece of music were analysed by principal component analysis (PCA). The present study hypothesized that a component shared within the melodies or bass lines and within major or minor keys represents a specific characteristic of TP distribution depending on voice part (i.e., melody and bass) and tonal mode (i.e., major and minor). Based on our previous papers [57], the criteria of the eigenvalue were set over 1. The first two components that contribute to each piece of music (i.e., the first and second highest cumulative contribution ratios), were adopted in Study 1. In Study 2, on the other hand, the first three components were adopted in order to verify the components of major and minor keys as well as those of the melody and bass lines. Furthermore, the *conditional entropy* (*H*(*AB*)) in the nth-order was calculated from the information content as follows:

$$H(B|A)=-\sum_{i}\sum_{j}P(ai)P(bj|ai)\;{log}_{2}\;P(bj|ai)\;(bits)$$ (3)

where *P(*bj|ai*)* is a conditional probability of the sequence *“ai bj”*. P(ai) is the probability of event ai occurring, and P(bj|ai) is the probability of bj occurring given that ai occurs previously (i.e., transitional probability). The conditional entropy is the sum of the bits and is regarded as the “*uncertainty*” of the transitional-probability distribution. The conditional entropy of each TP distribution was compared by correlation analysis. Statistical significance levels were set at *p* = 0.05 for all analyses.

## 3. Study 1: Ludwig van Beethoven

### 3.1. Results

#### 3.1.1. Retrieval of characteristics in the melody and the bass line in major and minor keys

**Fig 2:**
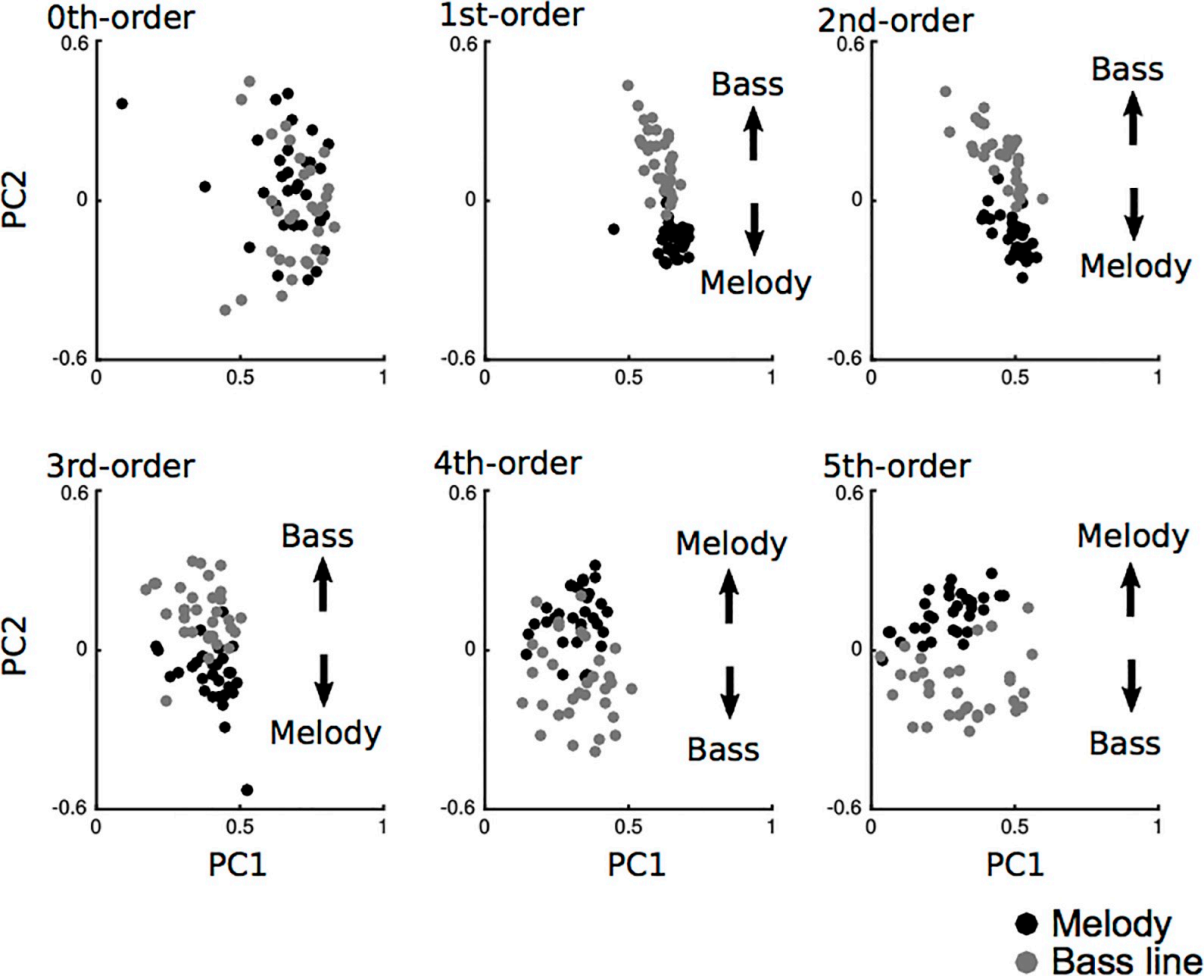
Principal component analysis scatter plots in melody (black) and bass line (grey) from zeroth- to fifth-order Markov models in Study 1 (Beethoven’ piano sonata). The horizontal axes and vertical axes represent principal components (PC) 1 and 2, respectively. Each dot represents a piece of music.

The transitional-probability matrices and the entropies in each piece of music are shown in Supporting Information 1 and 2, respectively. All of the results are shown in Table 1, Table 2, and Fig 2. In the zeroth-order model, the two components accounted for 51.18% of the total variance. All of the pieces of music except for No.20 scored higher than .37 on component 1. This score represents the general component that is shared between the melody and the bass line. Component 2, in contrast, was unable to detect any shared characteristics between the melody and bass line. In the first-, second-, and third-order models, the two components accounted for 42.64%, 25.91%, and 18.56% of the total variance, respectively. All of the pieces of music scored higher than .44, .25, and .17 on component 1 in the first-, second-, and third-order models, respectively. These results represent the general component that is shared between the melody and the bass line. In component 2, on the other hand, the eigenvectors in the melody were generally higher than those in the bass lines. This represents the distinct components of the melody and bass lines. In the fourth- and fifth-order models, the two components accounted for 14.23% and 13.12% of the total variance, respectively. All of the pieces of music scored higher than .14 and .03 on component 1 in the fourth- and fifth-order models, respectively. These results represent the general component that is shared between the melody and the bass line. In component 2, the eigenvectors were generally lower in the melody than in the bass lines. This represents the distinct components of the melody and the bass line.

**Table 1:** The eigenvalue and percentages of variance and cumulative variance in Study 1 (Beethoven’s piano sonata).

Order	PC	Total	Variance *	Cumulative *
0th	1	30.030	46.922	46.922
	2	2.723	4.254	51.176
1st	1	25.357	39.620	39.620
	2	1.932	3.019	42.639
2nd	1	14.488	22.637	22.637
	2	2.095	3.274	25.911
3rd	1	9.852	15.394	15.394
	2	2.025	3.165	18.559
4th	1	7.044	11.006	11.006
	2	2.061	3.220	14.226
5th	1	6.575	10.273	10.273
	2	1.819	2.842	13.115

* percentage

PC = principal component

**Table 2:** The eigenvectors for the principal components in Study 1 (Beethoven’s piano sonata).

Order		0th		1st		2nd		3rd		4th		5th	
Component		PC 1	PC 2	PC 1	PC 2	PC 1	PC 2	PC 1	PC 2	PC 1	PC 2	PC 1	PC 2
Melody	No1	.688	-.088	.670	-.220	.525	-.291	.525	-.529	.383	.273	.180	.013
	No2	.664	.040	.657	-.204	.572	-.212	.486	-.124	.386	.320	.184	.178
	No3	.764	-.269	.658	-.134	.497	-.208	.406	-.173	.301	.244	.216	.124
	No4	.795	-.189	.665	-.124	.534	-.185	.442	-.178	.364	.214	.287	.149
	No5	.644	.088	.634	-.187	.522	-.132	.371	-.106	.297	.245	.270	.235
	No6	.669	.106	.671	-.124	.488	-.117	.439	-.030	.353	-.089	.331	.189
	No7	.375	.051	.621	-.226	.509	-.177	.472	-.164	.427	.145	.301	.067
	No8	.692	.048	.664	-.222	.529	-.212	.429	-.172	.340	.265	.318	.025
	No9	.682	.306	.685	-.173	.480	-.220	.420	-.052	.355	.195	.340	.129
	No10	.775	.120	.630	-.238	.487	-.132	.466	-.086	.391	.099	.285	.073
	No11	.730	.148	.711	-.212	.537	-.228	.525	-.529	.342	.260	.201	.132
	No12	.789	-.055	.660	-.105	.526	-.005	.440	.143	.272	-.094	.302	.165
	No13	.746	.142	.603	-.199	.510	-.099	.473	.018	.404	.017	.448	.203
	No14	.654	-.092	.628	.030	.442	.082	.218	.000	.146	-.014	.190	.082
	No15	.733	-.299	.670	-.146	.503	-.177	.441	-.203	.322	.236	.310	.211
	No16	.699	.059	.671	-.138	.541	-.172	.461	-.083	.375	.121	.459	.206
	No17	.532	-.174	.617	-.143	.385	-.070	.206	.014	.152	.058	.100	.033
	No18	.713	-.095	.634	-.060	.478	-.149	.370	-.020	.307	.124	.277	.269
	No19	.667	.405	.658	-.181	.551	-.217	.446	-.288	.340	.216	.067	.069
	No20	.088	.366	.445	-.105	.414	-.070	.349	-.045	.246	.124	.040	-.038
	No21	.621	-.013	.637	-.086	.492	-.058	.423	.005	.409	.072	.331	.193
	No22	.624	.379	.640	-.002	.404	.001	.364	.079	.350	-.103	.418	.291
	No23	.631	-.280	.634	-.175	.487	-.085	.371	-.025	.320	.033	.333	.067
	No24	.558	.229	.625	-.116	.417	-.121	.255	-.097	.172	.102	.058	.068
	No25	.664	.192	.628	-.004	.389	-.056	.284	-.087	.215	.160	.150	.087
	No26	.728	.020	.649	-.138	.520	-.208	.408	-.092	.333	.097	.390	.202
	No27	.639	.155	.689	-.097	.448	-.054	.336	-.063	.216	.106	.348	.186
	No28	.779	-.074	.708	-.134	.493	-.201	.379	-.153	.257	.139	.208	.028
	No29	.688	-.088	.619	-.148	.512	-.199	.449	-.168	.403	.174	.389	.155
	No30	.806	.215	.691	-.164	.516	-.194	.407	-.052	.346	.142	.270	.209
	No31	.753	.264	.711	-.107	.530	-.107	.424	-.111	.273	.031	.198	.230
	No32	.578	.029	.704	-.137	.558	-.157	.461	-.139	.334	.085	.350	.163
Bass lines	No1	.791	.187	.634	.251	.479	.193	.331	.072	.164	-.081	.152	-.103
	No2	.783	-.023	.621	.212	.497	.220	.406	.054	.349	.051	.204	-.130
	No3	.631	-.038	.646	-.018	.524	.045	.420	.024	.334	.072	.183	-.085
	No4	.828	-.103	.647	.116	.501	.073	.403	.110	.349	-.167	.330	-.220
	No5	.742	.117	.644	.121	.475	.230	.304	.152	.163	.024	.077	-.171
	No6	.753	-.024	.638	.240	.509	.148	.418	.149	.357	-.126	.268	-.086
	No7	.679	-.297	.631	.064	.509	.157	.393	.054	.396	-.041	.307	-.076
	No8	.763	-.184	.594	.269	.377	.295	.289	.236	.259	-.242	.307	-.241
	No9	.671	.229	.552	.308	.351	.207	.360	.329	.387	-.384	.485	-.113
	No10	.690	-.051	.555	.111	.395	.198	.306	.120	.257	.092	.114	.016
	No11	.708	.160	.639	.058	.518	.047	.501	.120	.508	-.147	.524	-.212
	No12	.738	-.235	.568	.267	.389	.349	.333	.333	.293	-.240	.340	-.305
	No13	.801	.013	.653	.169	.470	.171	.433	.319	.400	-.339	.506	-.231
	No14	.765	-.039	.531	.362	.272	.261	.199	.256	.195	-.320	.274	-.242
	No15	.782	-.219	.585	.138	.477	.210	.352	.151	.308	-.183	.299	-.162
	No16	.772	-.111	.592	.207	.464	.178	.435	.189	.422	-.198	.499	-.190
	No17	.640	-.220	.545	.213	.360	.313	.210	.251	.133	-.196	.141	-.289
	No18	.642	-.360	.574	.203	.393	.166	.307	.156	.326	-.158	.370	.074
	No19	.655	.279	.625	.039	.473	.037	.245	-.188	.181	.181	.035	-.023
	No20	.535	.452	.499	.438	.258	.414	.172	.228	.198	-.206	.333	-.214
	No21	.672	-.232	.643	.152	.488	.210	.407	.195	.377	-.102	.412	-.225
	No22	.606	.256	.539	.231	.355	.186	.306	.070	.236	-.053	.193	-.294
	No23	.505	-.374	.647	.078	.502	.228	.434	.222	.448	-.251	.561	-.015
	No24	.732	-.230	.569	.192	.429	.117	.394	.046	.338	.205	.172	-.030
	No25	.504	.379	.584	.310	.391	.293	.243	.140	.201	-.007	.204	-.157
	No26	.768	-.038	.618	.082	.444	.179	.389	.282	.451	-.321	.533	-.159
	No27	.722	.101	.646	.033	.419	.213	.336	.201	.307	-.360	.417	.094
	No28	.674	-.066	.633	-.057	.506	-.026	.393	-.033	.255	.105	.103	-.091
	No29	.447	-.414	.574	-.006	.518	.005	.459	.007	.451	.006	.483	-.101
	No30	.609	-.003	.653	.010	.513	.024	.466	.085	.440	-.120	.368	-.251
	No31	.807	.043	.682	.062	.592	.010	.483	.070	.433	-.101	.545	.158
	No32	.608	-.194	.605	.083	.504	.106	.459	.118	.417	-.149	.372	-.248

#### 3.1.2. Correlation analysis

**Fig 3:**
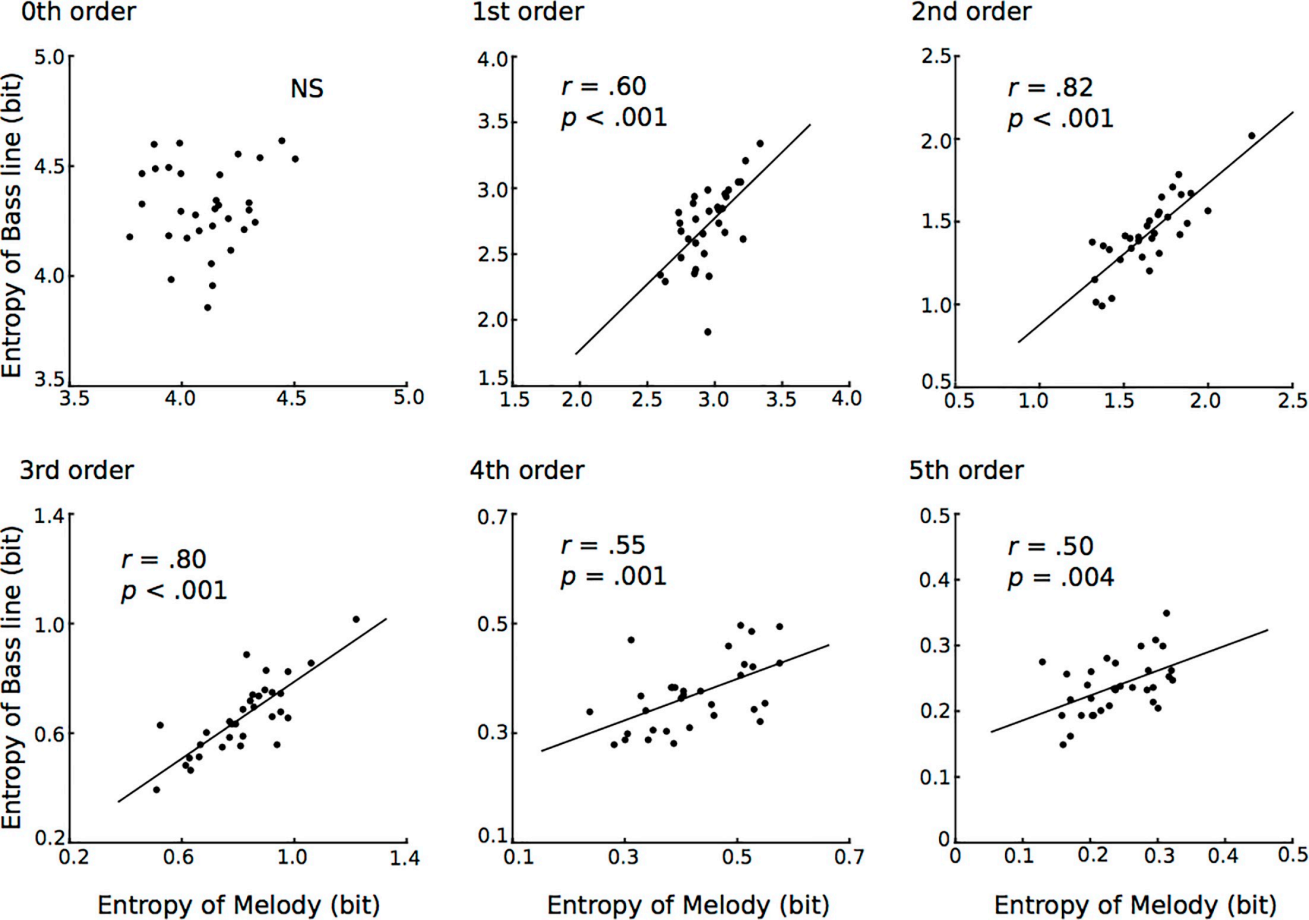
The correlation analysis of conditional entropy between the melody and bass line based on zeroth- to fifth-order Markov models in Study 1 (Beethoven’s piano sonata).

All of the results in the correlation analysis are shown in Fig 3. In first- to fifth-order TP distributions, the conditional entropies of the melody were significantly related to those of the bass line (1st: r = .60, *p* < 0.001; 2nd: r = .82, *p* < 0.001; 3rd: r = .80, *p* < 0.001; 4th: r = .55, *p* = 0.001; 5th: r = .50, *p* = 0.004).

### 3.2. Discussion

This study examined how zeroth- to fifth-order TP distributions (Markov models) and the conditional entropies in the melody and bass line correlate and interact with each other in all movements of the piano sonatas by Ludwig van Beethoven (No.1 in F minor, Op.2-1 to No.32 in C minor, Op.111, composed 1795–1822). First, we investigated how the statistical characteristics of the melody and bass line can be extracted in each order Markov model using principal component analysis. It was hypothesized that there were general statistical characteristics shared between the melody and bass line as well as specific statistical characteristics that were unique to each melody and bass line based on each order model. Thus, TP distribution in the zeroth-order Markov model detected a general component that is shared between the melody and bass line, whereas those in the first- to fifth-order Markov models detected specific components that are unique to each melody and bass line (Fig 2). These results suggest that specific statistical characteristics in each melody and bass line can be disclosed in higher-order but not in zeroth-order statistical models. From the psychological and neurophysiological viewpoints of SL in the brain, higher-order but not lower-order statistical knowledge of the melody and bass line are partially independent of each other.

Second, we investigated the relationships of conditional entropies between the melody and bass line in each order Markov model using correlation analysis. It was hypothesized that the correlation of the variability in the entropy between the melody and bass line depends on the order of TP distribution. The results suggest that the correlation of conditional entropies between the melody and bass line could be detected in the first- to fifth- but not zeroth-order Markov models. They may suggest a correlation in the variability of entropies between the melody and bass line in higher-order TP distributions. This may suggest that the correlation between the melody and bass line depends on the length of the sequence. Compared to the zeroth-order model, the higher models could essentially construct a musical phrase. Thus it is possible that the analysis of an entire musical phrase may strengthen the perceived connection between the melody and bass line. In psychological and computational studies related to SL, predictive coding, and information theory, entropy has been interpreted as the average degree of surprise associated with an outcome [33]. Entropy has also been used to verify the validity of statistical models in music [34–37]. The present study detected that the entropy of the melody is correlated with that of the bass line in higher-order statistical models. This may suggest that higher-order but not lower-order statistical knowledge of the melody and the bass line are partially dependent on each other. This hypothesis seems plausible given what we know about musical properties. In general, musical constraints such as harmony and musical key control phrasing of each melody and bass line. For example, if a five-tone melody is made up of C sharp, F sharp, and D (Fig 1, fourth-order), it controls a harmony or key (e.g., the A major, F-sharp minor, D major, or B minor keys), and the concurrent bass line also follows the same key or harmony. In contrast, a two-tone sequence with a semi- or whole-tone interval, which can be coded in a first-order model, is insufficient to establish a harmony, musical key, and phrase, unlike longer sequences. It is worth noting, however, that a pianist often picks up his or her hands as a phrase ends and restarts a new phrase, resulting in unpredictable jumps in pitch interval. Thus, we cannot exclude the possibility that the findings of the present study could simply be associated with texture and phrasing in music rather than melody and bass patterning itself. Further study will be needed to verify the relationships between musicological texture and statistical pattern with regard to entropy in several orders of TP distributions. In summary, this study may suggest that the SL of the melody and bass line correlate with and are partly independent of each other in terms of TP distribution. These findings may also be in agreement with the hypothesis in neural studies that the SL of the melody and bass line interact with and are partly independent of each other [29,61,65]. In the present studies, it was expected that this would occur based on some very specific findings in the neuroscience literature, but a previous neural study also suggested that SL could be modulated by music-specific features such as tonal mode and key [29]. Therefore, our next study will investigate how the tonalities of keys govern statistical distributions and the interaction between the melody and bass line.

## 4. Study 2: Johann Sebastian Bach

### 4.1. Results

#### 4.1.1. Retrieval of characteristics in the melody and bass lines in major and minor keys

**Fig 4:**
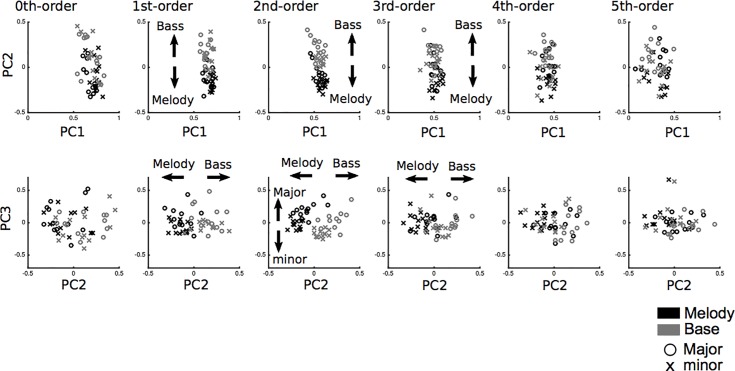
The correlation analysis of conditional entropy between the melody (black) and bass line (grey) in major and minor keys based on zeroth- to fifth-order Markov models in Study 2 (Bach’s Well-Tempered Clavier).

All of the results are shown in Table 3, Table 4, and Fig 4. In the zeroth- to fifth-order models, the three components accounted for 58.71%, 50.03%, 37.41%, 31.31%, 24.14%, and 15.94% of the total variance, respectively. All of the pieces of music scored higher than 0 on component 1, which represents the general component that is shared among all of the pieces of music. In component 2, in the first-, second-, and third-order models, the eigenvectors of the bass line were generally higher than those of the melody, representing the distinct components of the melody and the bass line. In component 3, in the second-order model, the eigenvectors of major keys were generally higher than those of minor keys, representing the various components of major and minor keys.

**Table 3:** The eigenvalue and percentages of variance and cumulative variance in Study 2 (Bach’s Well-Tempered Clavier).

Order	PC	Total	Variance *	Cumulative *
0th	1	23.78	49.53	49.53
	2	2.28	4.74	54.27
	3	2.13	4.44	58.71
1st	1	21.26	44.28	44.28
	2	1.58	3.29	47.57
	3	1.18	2.46	50.03
2nd	1	15.14	31.54	31.54
	2	1.53	3.18	34.72
	3	1.30	2.70	37.41
3rd	1	12.51	26.06	26.06
	2	1.35	2.81	28.87
	3	1.17	2.44	31.31
4th	1	8.91	18.56	18.56
	2	1.41	2.93	21.49
	3	1.27	2.65	24.14
5th	1	4.89	10.18	10.18
	2	1.40	2.92	13.10
	3	1.36	2.83	15.94

* percentage

PC = principal component

**Table 4:** The eigenvectors for the principal components in Study 2 (Bach’s Well-Tempered Clavier).

Order			0th			1st			2nd			3rd			4th			5th		
Component			PC 1	PC 2	PC 3	PC 1	PC 2	PC 3	PC 1	PC 2	PC 3	PC 1	PC 2	PC 3	PC 1	PC 2	PC 3	PC 1	PC 2	PC 3
Melody	Major	C	0.72	-0.29	0.24	0.64	0.08	0.15	0.56	-0.08	0.19	0.47	-0.08	-0.01	0.34	-0.22	-0.03	0.14	-0.03	0.17
		Db	0.60	0.13	0.47	0.59	-0.07	0.44	0.46	0.15	0.28	0.50	0.16	0.43	0.45	0.21	-0.20	0.33	0.32	0.12
		D	0.65	0.10	-0.11	0.63	-0.03	-0.01	0.56	-0.06	0.00	0.48	-0.09	0.01	0.41	0.18	0.21	0.34	0.12	-0.10
		Eb	0.65	-0.06	-0.03	0.69	-0.15	0.00	0.59	-0.07	0.22	0.48	-0.21	0.09	0.48	0.01	0.15	0.42	-0.11	-0.15
		E	0.74	-0.21	-0.12	0.65	-0.16	-0.03	0.59	-0.13	0.24	0.57	-0.04	0.06	0.50	-0.05	-0.09	0.40	0.18	0.16
		F	0.67	-0.27	0.21	0.70	-0.13	-0.03	0.59	-0.23	0.15	0.53	-0.26	-0.13	0.45	-0.09	0.14	0.35	-0.09	0.02
		Gb	0.75	-0.25	0.33	0.69	-0.22	0.19	0.53	-0.01	0.28	0.51	0.00	0.09	0.37	0.12	-0.02	0.07	-0.02	0.00
		G	0.71	-0.22	0.00	0.61	-0.32	0.23	0.48	0.10	0.42	0.47	0.24	0.08	0.49	0.25	0.11	0.38	0.20	-0.07
		Ab	0.66	0.15	0.52	0.72	-0.22	0.05	0.62	-0.08	0.15	0.53	0.00	0.06	0.45	0.01	-0.08	0.35	0.02	-0.14
		A	0.69	-0.32	-0.03	0.71	0.00	-0.21	0.57	-0.14	0.07	0.53	0.00	0.10	0.38	-0.03	-0.05	0.25	0.17	0.01
		Bb	0.61	-0.03	-0.35	0.61	-0.14	0.14	0.52	-0.15	0.13	0.51	0.04	-0.21	0.46	0.01	-0.32	0.44	-0.02	-0.14
		B	0.73	-0.18	-0.10	0.69	-0.21	-0.06	0.59	-0.19	0.20	0.60	-0.08	0.12	0.51	0.01	-0.07	0.39	-0.21	0.09
	minor	C	0.72	0.14	0.22	0.71	-0.25	0.03	0.56	-0.23	0.03	0.45	-0.26	0.29	0.32	-0.01	0.14	0.14	0.00	0.03
		Db	0.75	-0.14	-0.08	0.71	-0.03	-0.09	0.61	-0.16	-0.11	0.51	-0.26	0.11	0.46	-0.11	0.15	0.27	0.17	-0.10
		D	0.63	0.19	-0.16	0.63	-0.15	0.05	0.49	-0.12	0.03	0.47	0.04	-0.08	0.33	-0.11	-0.02	0.17	-0.11	0.07
		Eb	0.78	0.01	0.16	0.69	-0.08	-0.12	0.62	-0.22	-0.03	0.60	-0.18	-0.07	0.52	-0.18	0.01	0.44	-0.06	-0.03
		E	0.78	0.21	-0.15	0.69	-0.28	-0.16	0.56	-0.16	-0.06	0.53	-0.09	-0.08	0.52	-0.14	-0.12	0.40	-0.31	-0.04
		F	0.68	-0.17	0.32	0.68	-0.27	0.08	0.56	-0.26	0.10	0.50	-0.25	-0.10	0.41	-0.12	0.26	0.39	-0.05	0.06
		Gb	0.75	-0.03	0.15	0.74	-0.12	-0.14	0.56	-0.23	-0.03	0.53	-0.17	0.03	0.44	-0.15	-0.06	0.35	-0.24	-0.02
		G	0.75	-0.21	-0.01	0.72	-0.07	0.02	0.54	-0.19	0.03	0.48	-0.34	-0.01	0.37	-0.37	0.16	0.20	-0.06	0.67
		Ab	0.82	-0.32	0.16	0.70	-0.16	-0.16	0.61	-0.20	-0.12	0.57	-0.15	0.16	0.43	-0.19	0.15	0.22	-0.16	0.16
		A	0.78	-0.17	-0.16	0.72	-0.07	-0.14	0.64	-0.15	-0.11	0.58	-0.08	-0.15	0.50	-0.24	-0.08	0.42	-0.22	-0.02
		Bb	0.74	-0.26	-0.03	0.68	-0.08	-0.16	0.64	-0.14	0.02	0.59	-0.12	0.06	0.47	-0.32	-0.04	0.35	-0.33	0.10
		B	0.76	-0.05	-0.22	0.70	-0.20	-0.16	0.60	-0.30	-0.11	0.54	-0.20	-0.15	0.44	-0.10	0.07	0.38	-0.05	-0.02
Bass	Major	C	0.80	-0.19	-0.01	0.58	0.25	0.17	0.54	0.22	-0.07	0.52	0.24	-0.04	0.41	0.02	-0.28	0.22	0.11	0.20
lines		Db	0.65	0.34	0.04	0.57	0.17	0.48	0.46	0.36	0.12	0.52	0.23	0.37	0.40	0.20	-0.28	0.24	0.31	0.18
		D	0.63	0.36	0.12	0.58	0.36	0.17	0.55	0.28	-0.19	0.45	0.19	-0.05	0.39	0.28	0.24	0.25	0.17	-0.08
		Eb	0.76	0.05	-0.17	0.63	0.16	0.01	0.53	0.08	-0.09	0.46	0.02	-0.04	0.41	0.13	0.28	0.28	-0.05	-0.17
		E	0.58	-0.16	0.17	0.68	0.30	-0.13	0.63	0.25	0.06	0.58	0.19	-0.01	0.47	0.28	0.18	0.29	0.44	-0.03
		F	0.54	-0.07	-0.03	0.64	0.16	-0.21	0.57	0.05	-0.21	0.51	-0.09	-0.14	0.44	0.11	-0.07	0.32	-0.01	-0.05
		Gb	0.62	0.33	-0.22	0.63	-0.01	0.29	0.51	0.30	0.14	0.44	0.25	0.05	0.29	0.16	0.11	0.15	0.09	-0.04
		G	0.63	0.33	0.12	0.62	0.01	0.32	0.42	0.41	0.36	0.39	0.42	0.10	0.39	0.36	0.00	0.21	0.20	-0.03
		Ab	0.75	0.07	-0.30	0.68	-0.03	0.07	0.57	0.16	0.10	0.53	0.18	-0.09	0.47	0.11	-0.16	0.40	-0.04	-0.24
		A	0.79	0.11	0.17	0.66	0.25	-0.07	0.50	0.27	0.03	0.50	0.24	0.11	0.45	0.20	-0.15	0.33	0.26	-0.12
		Bb	0.56	0.37	-0.10	0.62	0.08	-0.04	0.52	0.04	0.05	0.50	0.03	-0.27	0.38	0.08	-0.27	0.35	-0.07	-0.17
		B	0.83	-0.09	-0.02	0.71	0.20	-0.02	0.60	0.17	0.04	0.57	0.23	-0.07	0.50	0.25	-0.09	0.49	0.20	-0.05
	minor	C	0.73	-0.12	-0.19	0.62	0.13	0.03	0.52	0.12	-0.07	0.35	-0.03	0.42	0.24	0.17	0.37	0.19	0.24	0.03
		Db	0.67	0.10	-0.23	0.72	0.21	-0.06	0.61	0.02	-0.22	0.51	-0.01	-0.08	0.47	-0.01	0.10	0.26	0.07	0.09
		D	0.70	0.13	0.09	0.63	0.10	0.05	0.55	0.16	-0.11	0.49	0.13	-0.22	0.35	0.01	-0.14	0.19	-0.04	-0.04
		Eb	0.79	-0.20	0.09	0.70	0.06	0.02	0.57	0.00	-0.16	0.56	0.09	-0.22	0.55	0.01	0.02	0.46	0.07	-0.01
		E	0.71	0.40	-0.07	0.66	-0.10	-0.16	0.52	0.00	-0.08	0.55	0.06	-0.08	0.47	-0.15	-0.13	0.33	-0.32	-0.07
		F	0.54	0.46	0.20	0.62	0.16	-0.01	0.60	0.10	-0.25	0.51	0.02	-0.08	0.45	0.13	0.31	0.39	0.01	-0.03
		Gb	0.78	-0.16	-0.27	0.68	0.31	-0.01	0.59	0.12	-0.18	0.53	0.12	-0.08	0.46	0.01	-0.15	0.36	-0.20	-0.12
		G	0.71	-0.04	0.04	0.69	0.21	-0.08	0.55	0.16	-0.19	0.41	0.04	-0.12	0.32	-0.31	0.20	0.14	0.00	0.64
		Ab	0.58	0.40	0.40	0.62	0.10	-0.04	0.54	0.10	-0.21	0.47	-0.15	0.20	0.35	-0.13	0.07	0.11	-0.16	0.08
		A	0.67	0.12	-0.40	0.67	0.07	-0.12	0.60	0.04	-0.22	0.53	0.08	-0.05	0.46	-0.21	-0.03	0.37	-0.02	0.05
		Bb	0.76	0.12	-0.20	0.69	0.39	-0.13	0.63	0.12	-0.17	0.58	-0.05	0.02	0.49	-0.11	-0.07	0.37	-0.07	0.21
		B	0.70	0.17	-0.27	0.70	-0.04	-0.11	0.58	0.07	-0.14	0.44	0.16	-0.20	0.40	0.08	-0.06	0.23	0.06	-0.01

#### 4.1.2. Correlation analysis

**Fig 5:**
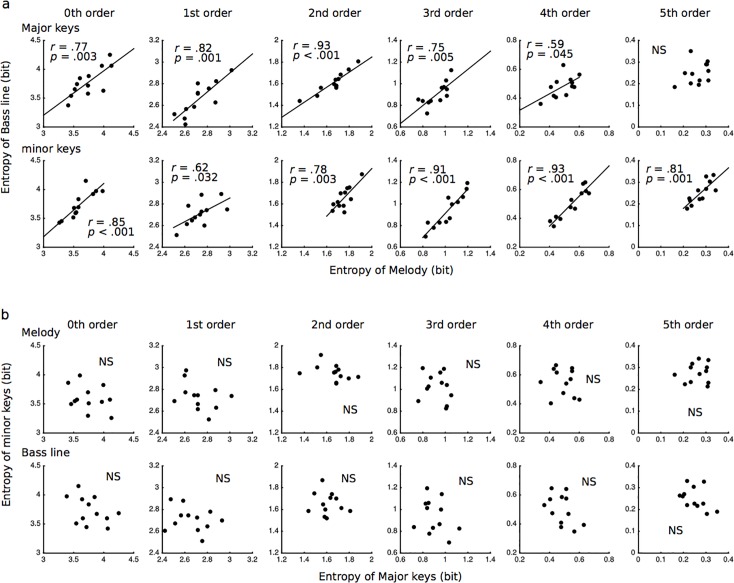
The correlation analysis of conditional entropy between the melody and bass line (**a**), and between major and minor keys (**b**), based on zeroth- to fifth-order Markov models in Study 2 (Bach’s Well-Tempered Clavier).

All of the results in the correlation analysis are shown in Fig 5. In the zeroth-, second-, and third-order TP distributions, the conditional entropies of the melody were strongly (0.7≦|r|<1.0) related to those of the bass line (zeroth: major: r = .77, *p* = 0.003; minor: r = .85, *p* < 0.001, second: major: r = .93, *p* < 0.001; minor: r = .78, *p* = 0.003, third: major: r = .75, *p* = 0.005; minor: r = .91, *p* < 0.001; Fig 5A). In first-order TP distributions, the conditional entropies of the melody in major keys were strongly related while those in minor keys were moderately (0.4≦|r|<0.7) related to those of the bass line (major: r = .82, *p* = 0.001; minor: r = .62, *p* = 0.063). In fourth-order TP distributions, the conditional entropies of the melody in major keys were moderately related while those in minor keys were strongly related to those of the bass line (major: r = .59, *p* = 0.045; minor: r = .93, *p* < 0.001). In fifth-order TP distributions, the conditional entropies of the melody were strongly related to those of the bass line in minor keys (r = .81, *p* = 0.001), whereas no significant correlation was detected in major keys. No significant correlation was detected between major and minor keys (Fig 5B).

### 4.2. Discussion

In Study 2, using Johann Sebastian Bach’s Well-Tempered Clavier, BWV 846–893, which has preludes and fugues in all 24 major and minor keys, we investigated the interaction between the zeroth- to fifth-order TP distributions (Markov models) and the conditional entropies in the melody and bass line. First, the manner in which the statistical characteristics of the melody and bass line in each of the major and minor keys could be extracted in each order Markov model was investigated using principal component analysis. It was hypothesized that there were general statistical characteristics shared between the melody and the bass line and between the major and minor keys, as well as specific statistical characteristics that were unique to each melody and bass line and to each major or minor key. Additionally, it was hypothesized that the detectability of these characteristics depends on the tonalities of the keys and the order of TPs [63]. Thus, TP distribution in each order Markov model detected general components that are shared between the melody and bass line and between major and minor keys (Fig 4). The first- to third-order Markov models detected specific components that are unique to each melody and bass line. The second-order Markov models detected specific components that are unique to each major and minor key**1**. These results suggest that statistical characteristics specific to each melody and bass line can be disclosed in first- to third-order models. Second, we investigated the relationships of conditional entropies between the melody and bass line and between major and minor keys in each order Markov model using correlation analysis. It was hypothesized that the correlation of variability in the entropies between the melody and bass line depends on the order of TP distribution and tonal mode. The results suggested that the correlation of conditional entropies between the melody and bass line could be detected in the first- to fifth- but not zeroth-order Markov models. These results suggest that the variability of entropies is correlated with the melody and bass line in each order TP distribution. Considering the psychological and computational viewpoints on entropy [34], the present findings that the entropies of the melody are correlated with those of the bass line suggest that statistical knowledge of the melody and bass line, but not of major and minor keys (Fig 5B), are partially dependent on each other. In summary, this study suggested that SL of the melody and SL of the bass line correlate with and are partly independent of each other. Thus, humans’ statistical knowledge of melodies and bass lines may be derived from their pairing with some noise in compositional systems.

## 5. General discussion

### 5.1. Statistical characteristics of melodies and bass lines

The present studies investigated how TP distributions and the conditional entropy of the melody and bass line interact with each other, using the highest and lowest pitches in Beethoven’s piano sonatas (Study 1) and Johann Sebastian Bach’s Well-Tempered Clavier (Study 2). Our findings were similar for the two composers. First, TP distribution in each model showed a general component (component 1) that is shared between the melody and bass line. Second, TP distribution in the first- and second- but not zeroth-order models detected specific components (component 2) that were unique to each melody and bass line. These results suggest that statistical characteristics specific to each melody and bass line can be disclosed in higher-order but not in zeroth-order statistical models. From the psychological and neurophysiological viewpoints of SL in the brain, higher-order but not lower-order statistical knowledge of the melody and bass line are partially independent of each other. Additionally, Study 2 also detected specific components (component 3) that are unique to each major and minor key as well as to the melody and bass line (Fig 4). Thus, the results suggest that a second-order Markov model (i.e., trigram model) may have the advantage of being able to extract statistical characteristics based on the tonalities of keys and voice parts. From a psychological viewpoint, a composer’s specific statistical knowledge of the melody and bass lines in music may be expressed in higher-order rather than zeroth-order TP distributions. It is of note, however, that the present studies investigated statistical characteristics in music belonging to only two corpora without taking any psychological or neurological measurements and did not directly demonstrate statistical knowledge of music in the composers. A previous study reported computational validation against a ground truth of human cognition by examining whether the output of computational modelling aligned with human assessments or behaviour [21]. Thus, it may be doubtful to claim that neurodynamics can be represented by TP distribution and entropy. Furthermore, the present studies might not prove the existence of a general musical phenomenon because of the small corpora, and there might be other possible explanations for our results. For instance, it might have been an intentional plan on the part of the composers to compose music based on the statistics of melodies and bass lines. Furthermore, it has been suggested that humans’ ability to generate random sequences of numbers [66] is associated with creativity [67]. The possibility that the findings in the present studies do not necessarily reflect the composers’ statistical learning cannot be excluded. Thus, it remains possible that the findings of these studies showed compositional tendencies that are present in the examined corpus but may not be inherent to cognitive function in the human brain. Future studies are required to investigate the phenomenon of music learning through experimentation and direct comparison of computational and neurophysiological results.

### 5.2. Relationships of entropy between the melody and the bass line

In the fields of computational and informatics studies, entropy has been used to verify the validity of computational models including SL in music (e.g., [34]). A computational model with lower entropy indicates greater predictability. Additionally, in the fields of neuroscience and psychology, entropy has been interpreted as the average degree of surprise associated with outcomes based on predictions in the brain [32]. Thus, both computational researchers and psychologists agree that entropy in the framework of statistical learning can highlight some of the statistical information that is available to music learners. Based on these studies, the present studies expected the variation of entropy in music to partially reflect typical patterns in musical expression associated with statistical knowledge. The results suggested that the correlation of conditional entropies between the melody and the bass line could be detected in some Markov models for both composers. This suggests that the variability in entropy is correlated between the melody and the bass line in TP distributions. In psychological and computational studies related to SL, predictive coding, and information theory, entropy has been interpreted as the average degree of surprise associated with an outcome [33]. Based on neurophysiological theories, when the brain encodes TP distributions in musical sequences, a next tone can be expected. Based on this processing, a neurophysiological response to predictable external stimuli can be inhibited to ensure efficiency and low entropy of neural processing[68][69] [70]. Thus, the correlation between the melody and the bass line suggests that statistical knowledge of the melody and that of the bass line interact with each other. However, the results of Study 2 also suggest that the correlations of TP distributions and the entropies between the melody and the bass line partly depend on tonalities (i.e., major and minor keys). In the second-order model, the specific characteristics of TP distributions could be detected in major and minor keys of each melody and bass line. Additionally, the correlation of entropy between the melody and the bass line in the fifth-order model could be detected in minor keys but not in major keys. This may be because there is more variation in minor keys than in major ones, as the sixth and seventh scale degrees are more variable in minor keys than in major keys [71]. Another possibility is that, as previous studies have reported, SL of the melody and SL of the bass line interact with and are partly independent of each other [61,65], and SL can be modulated by music-specific features such as tonal mode and key [29]. The present studies may be in agreement with these previous neurophysiological findings. Thus, neurophysiological and computational findings may partially share SL. On the other hand, the computational approaches in the present study did not consider pitch intervals between the melody and the bass line, although this is important information in the establishment of harmony and in the prediction of when the melodies and bass lines will act similarly and when they will act differently. In this study, the two lines were analysed as independent information and compared in order to explore whether the entropy levels of these lines are correlated with each other. Our studies suggest that statistical knowledge, which has been demonstrated by several neurophysiological studies, is mentally expressed in music composition. Future studies are required to investigate the neural basis underlying the mental expression of acquired statistical knowledge by directly comparing computational and neurophysiological results in an experiment. The present studies may propose novel methodologies that can be used to evaluate the statistical knowledge of a composer via interdisciplinary approaches that include informatics, musicology, and psychology.

## Supporting information

S1 Table Transitional-probability matrices in all pieces of music. (XLSX)

S2 Table Entropies in all pieces of music. (XLSX)
